# 

**Published:** 1871-11

**Authors:** 


### BOOK NOTICES.

"The Guide Board" to Health, Happiness, and a Cheerful Old Age, is the same book which an English lady was so often found to be carrying about under her arm in the south of Europe last winter. A gentleman as tall as a cupola, that is, seven feet seven inches, writes us to-day: "I bought the 'Guide Board,' and wish I had seen it years ago, and had had sense enough to take some of the good advice in it" This is a book of seven hundred pages, large type, at various prices, from four to seven dollars, published by D. E. Fiske & Co., Springfield, Mass., H. N. McKinney & Co., 16 North Seventh St., Philadelphia, and F. A. Hutchinson & Co., 502 North Sixth St., St. Louis, Mo. It contains a very large amount of valuable, practical reading, suitable for all ages, classes, and conditions.

"The Last Knight," a Romance Garland, published by Hurd & Houghton. From the German of Anastatius Grün. Translated with Notes by John O. Sargent. In one volume, 4to. Price $2.50. "The Last Knight" is a series of ballads founded on incidents in the life of the Emperor Maximilian I., and forming a national poem which has given the author his chief and deserved celebrity.

